# Provisional Mortality Data — United States, 2021

**DOI:** 10.15585/mmwr.mm7117e1

**Published:** 2022-04-29

**Authors:** Farida B. Ahmad, Jodi A. Cisewski, Robert N. Anderson

**Affiliations:** 1 National Center for Health Statistics, CDC, Hyattsville, Maryland.

The CDC National Center for Health Statistics’ (NCHS) National Vital Statistics System (NVSS) collects and reports annual mortality statistics using U.S. death certificate data. Because of the time needed to investigate certain causes of death and to process and review death data, final annual mortality data for a given year are typically released 11 months after the end of the calendar year. Provisional data, which are based on death certificate data received but not fully reviewed by NCHS, provide an early estimate of deaths before the release of final data. NVSS routinely releases provisional mortality data for all causes of death and for deaths involving COVID-19.* This report presents an overview of provisional U.S. mortality data for 2021, including a comparison of death rates for 2020 and 2021. In 2021, approximately 3,458,697 deaths^†^ occurred in the United States. From 2020 to 2021, the age-adjusted death rate (AADR) increased by 0.7%, from 835.4 to 841.6 per 100,000 standard population. COVID-19 was reported as the underlying cause or a contributing cause in an estimated 460,513 (13.3%) of those deaths (111.4 deaths per 100,000). The highest overall death rates by age occurred among persons aged ≥85 years, and the highest overall AADRs by sex and race and ethnicity occurred among males and non-Hispanic American Indian or Alaska Native (AI/AN) and non-Hispanic Black or African American (Black) populations. COVID-19 death rates were highest among persons aged ≥85 years, non-Hispanic Native Hawaiian or other Pacific Islander (NH/OPI) and AI/AN populations, and males. For a second year, the top three leading causes of death by underlying cause were heart disease, cancer, and COVID-19. Provisional death estimates provide an early indication of shifts in mortality trends and can guide public health policies and interventions aimed at reducing mortality directly or indirectly associated with the pandemic and among persons most affected, including persons who are older, male, or from certain race and ethnic minority groups.

This report analyzed provisional NVSS death certificate data for deaths occurring among U.S. residents in the United States during January–December 2021. NCHS tabulated the number and rates of overall deaths and COVID-19 deaths by age, sex, and race and ethnicity (categorized as Hispanic, non-Hispanic White [White], non-Hispanic Black, non-Hispanic Asian [Asian], non-Hispanic AI/AN, non-Hispanic NH/OPI, non-Hispanic persons of more than one race [multiracial], and unknown). NCHS coded the causes of death according to the *International Classification of Diseases, Tenth Revision*, which details disease classification and the designation of underlying cause of death (*1*,*2*). COVID-19 death counts and rates include deaths for which confirmed or presumed COVID-19 is listed on the death certificate as an underlying or contributing cause of death.^§^ COVID-19 was the underlying cause for approximately 90% (415,399), and a contributing cause of death for the remaining 10% (45,114) of COVID-19–associated deaths in 2021 (*3*). Leading causes of death were ranked by counts of underlying cause of death (*4*). NVSS data in this report exclude deaths among residents of U.S. territories and foreign countries.^¶^ Age was unknown for 73 (<0.01%) decedents, and race and ethnicity were unknown for 8,382 (0.24%) decendents. There were no records with unknown sex. To describe the trend in deaths during one year, the number of deaths were calculated for each week from all causes and from COVID-19 as an underlying or contributing cause. AADRs were calculated for deaths by sex and race and ethnicity. Crude death rates were calculated by age. Provisional death counts and rates for 2021 were compared with final 2020 data (*5*). The population data used to estimate death rates presented in this report are July 1, 2021, monthly postcensal population estimates based on the 2010 decennial census (*6*). R statistical software (version 4.0.3; The R Foundation) was used to conduct all analyses. This activity was reviewed by CDC and was conducted consistent with applicable federal law and CDC policy.**

In 2021, approximately 3,458,697 deaths occurred in the United States (Table). The age-adjusted rate was 841.6 deaths per 100,000 standard population, an increase of 0.7% from 835.4 in 2020. The number of deaths peaked during the week ending January 16, 2021 (87,222) and during the week ending September 11, 2021 (73,466) (Figure 1). In 2021, overall death rates were lowest among persons aged 5–14 years (14.6) and highest among persons aged ≥85 years (13,826.2), similar to patterns in 2020 (Table). Death rates increased for most age groups from 2020 to 2021, except for persons aged 75–84 and ≥85 years. In 2021, AADRs were higher among males (1,011.0) than among females (694.6), similar to patterns in death rate by sex in 2020.

**TABLE:** Provisional* number and rate of total deaths and COVID-19–related deaths, by demographic characteristics — National Vital Statistics System, United States, 2020–2021

Characteristic	2020 No. (rate^†^)		2021 No. (rate^†^)	
	Total deaths	Deaths involving COVID-19^§^	Total deaths	Deaths involving COVID-19^§^
**Total**	**3,383,729 (835.4)**	**384,536 (93.2)**	**3,458,697 (841.6)**	**460,513 (111.4)**
**Age group, yrs**				
<1	19,582 (524.3)	52 (1.4)	19,724 (528.6)	155 (4.2)
1–4	3,529 (22.7)	25 (0.2)	3,773 (24.7)	63 (0.4)
5–14	5,623 (13.7)	68 (0.2)	5,955 (14.6)	175 (0.4)
15–24	35,816 (84.2)	612 (1.4)	38,234 (90.2)	1,634 (3.9)
25–34	73,486 (159.5)	2,609 (5.7)	82,039 (178.6)	6,971 (15.2)
35–44	10,4490 (248.0)	6,756 (16.0)	124,577 (292.0)	17,304 (40.6)
45–54	191,142 (473.5)	18,250 (45.2)	215,531 (538.5)	39,193 (97.9)
55–64	440,549 (1,038.9)	45,377 (107.0)	477,107 (1,131.8)	78,904 (187.2)
65–74	674,507 (2,072.3)	82,055 (252.1)	723,125 (2,148.2)	111,035 (329.9)
75–84	822,084 (4,997.0)	106,020 (644.4)	828,617 (4,882.6)	110,194 (649.3)
≥85	1,012,805 (15,210.9)	122,707 (1,842.9)	939,942 (13,826.2)	94,884 (1,395.7)
Unknown	116 (—)	5 (—)	73 (—)	1 (—)
**Sex**				
Female	1,613,845 (695.1)	175,818 (73.9)	1,623,861 (694.6)	202,006 (87.7)
Male	1,769,884 (998.3)	208,718 (117.0)	1,834,836 (1,011.0)	258,507 (140.0)
**Race/Ethnicity**				
Hispanic	305,708 (723.6)	69,069 (164.8)	315,128 (700.1)	72,685 (157.8)
White, non-Hispanic	2,484,072 (834.7)	232,555 (74.1)	2,545,602 (852.2)	303,595 (100.6)
Black, non-Hispanic	449,213 (1,119.0)	61,401 (154.8)	448,416 (1,081.2)	61,626 (146.8)
Asian, non-Hispanic	91,175 (457.7)	13,523 (67.2)	91,814 (439.6)	13,587 (64.0)
American Indian/Alaska Native, non-Hispanic	24,725 (1,036.2)	4,615 (190.8)	26,850 (1,088.5)	5,027 (198.5)
Native Hawaiian/other Pacific Islander, non-Hispanic	4,439 (821.3)	691 (123.5)	5,225 (916.5)	1,170 (199.8)
Multiracial, non-Hispanic	15,523 (376.9)	1,141 (31.9)	17,280 (399.5)	2,003 (49.8)
Unknown	8,874 (—)	1,541 (—)	8,382 (—)	820 (—)

* National Vital Statistics System provisional data for 2021 are incomplete. Data from December 2021 are less complete because of reporting lags. Data for 2020 are final. These data exclude deaths that occurred in the United States among residents of U.S. territories and foreign countries.

^†^ Deaths per 100,000 standard population. Age-adjusted death rates are provided overall and by sex and race and ethnicity.

^§^ Deaths with confirmed or presumed COVID-19 as an underlying or contributing cause of death, with *International Classification of Diseases, Tenth Revision* code U07.1.

**FIGURE 1:**
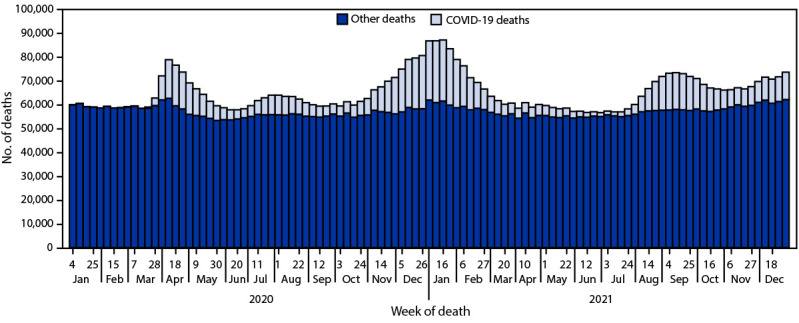
Provisional* number of COVID-19 deaths^†^ and other deaths, by week of death — National Vital Statistics System, United States, 2020–2021 * National Vital Statistics System provisional data for 2021 are incomplete. Data from December 2021 are less complete because of reporting lags. Data for 2020 are final. These data exclude deaths that occurred in the United States among residents of U.S. territories and foreign countries. ^†^ Deaths with confirmed or presumed COVID-19, coded to *International Classification of Diseases, Tenth Revision* code U07.1 as an underlying or contributing cause of death.

In 2021, COVID-19 was listed as the underlying or contributing cause of 460,513 deaths (111.4 per 100,000), an increase from 384,536 deaths (93.2) in 2020 (Table). In 2021, COVID-19 death rates were lowest among persons aged 1–4 (0.4) and 5–14 years (0.4) and highest among those aged ≥85 years (1,395.7). COVID-19 death rates increased from 2020 to 2021 for all age groups except for those aged ≥85 years. As with deaths overall, in 2021, the age-adjusted COVID-19–associated death rate among males (140.0) was higher than that among females (87.7).

AADRs differed by race and ethnicity. In 2021, overall AADRs were lowest among multiracial (399.5) and Asian persons (439.6) and highest among AI/AN (1,088.5) and Black persons (1,081.2). Similarly for 2021, COVID-19–associated death rates were lowest for multiracial (49.8) and Asian persons (64.0) and highest among NH/OPI (199.8) and AI/AN persons (198.5). Overall and COVID-19 death rates decreased for Hispanic, Black, and Asian persons from 2020 to 2021.

COVID-19, listed as the underlying cause in 415,399 deaths during 2021, ranked as the third leading underlying cause of death after heart disease (693,021 deaths) and cancer (604,553 deaths) (Figure 2). COVID-19 was the underlying cause for 13.3% of all deaths in 2021, increasing from 10.4% (350,831 deaths) in 2020. Unintentional injuries, the fourth leading cause of death in 2020 and 2021, increased from 200,955 in 2020 to 219,487 in 2021. Other leading causes of death maintained the same ranking from 2020 to 2021, except for chronic liver disease and cirrhosis and influenza and pneumonia. Chronic liver disease and cirrhosis, which was not among the 10 leading causes of death in 2020, was the ninth leading cause in 2021 with 56,408 deaths (51,642 deaths in 2020). Influenza and pneumonia, which was the ninth leading cause of death in 2020 (53,544 deaths), dropped out of the 10 leading causes in 2021 (41,835 deaths). 

**FIGURE 2:**
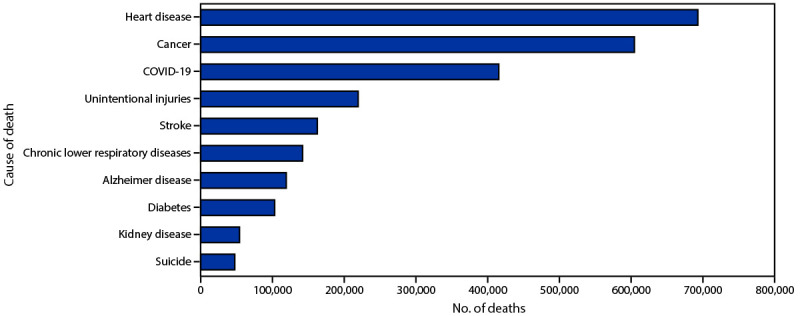
Provisional* number of leading underlying causes of death^†^— National Vital Statistics System, United States, 2021 * National Vital Statistics System provisional data are incomplete. Data from December are less complete because of reporting lags. Deaths that occurred in the United States among residents of U.S. territories and foreign countries were excluded. ^†^ Deaths for which COVID-19 was a contributing, but not the underlying cause of death are not included.

## Discussion

From 2020 to 2021, the age-adjusted U.S. death rate increased by 0.7%, from 835.4 to 841.6 per 100,000 standard population. In 2021, COVID-19 was the underlying or a contributing cause of death for 460,513 deaths (111.4 deaths per 100,000). COVID-19 death rates were highest among males, adults aged ≥85 years, and NH/OPI and AI/AN persons. The highest numbers of overall deaths and COVID-19 deaths occurred during January and September. COVID-19 was the third leading underlying cause of death in 2021, for the second year since the disease emerged (*6*).

Demographic patterns of mortality were similar in 2020 and 2021, but certain populations experienced shifts in death rates. Although the overall and COVID-19 death rate remained higher for persons aged ≥85 years than for all other age groups, death rates decreased for this age group from 2020 to 2021. Age-adjusted total and COVID-19 death rates remained high for the AI/AN population. Rates decreased for Asian, Hispanic, and Black populations and increased for NH/OPI, White, and AI/AN populations.

The year 2021 saw the highest death rate since 2003, with increases in many leading causes of death, including COVID-19 and unintentional injuries. Although COVID-19 death rates decreased for persons aged ≥85 years, age groups <75 years saw large increases from 2020 to 2021. Unintentional injury deaths were largely driven by drug overdose deaths, and likely contributed to the increased death rate in younger populations. In 2020, drug overdose death rates increased more for persons aged 15–64 years than for persons aged ≥65 years (*7*).

The findings in this report are subject to at least three limitations. First, data are provisional, and numbers and rates might change as additional information is received. Described changes in mortality trends might be underestimates. Second, timeliness of death certificate submission can vary by jurisdiction. As a result, the national distribution of deaths might be affected by the distribution of deaths reported from jurisdictions reporting later, which might differ from those in the United States overall. Finally, there is a higher potential for misclassification of certain categories of race (i.e., AI/AN or Asian) and Hispanic ethnicity reported on death certificates (*8*). Thus, death rates for some groups might be underestimated or overestimated.

Provisional death estimates can give researchers and policymakers an early projection of shifts in mortality trends and provide actionable information sooner than do the final mortality data, which are released approximately 11 months after the end of the data year. These data can guide public health policies and interventions aimed at reducing mortality directly or indirectly associated with the pandemic and among persons most affected, including persons who are older, male, or from certain race and ethnic minority groups.

**Summary**

**What is already known about this topic?**

COVID-19 was associated with approximately 460,000 deaths in the U.S. during January–December 2021.

**What is added by this report?**

The overall age-adjusted death rate increased by 0.7% in 2021 from 2020. Overall death rates were highest among non-Hispanic American Indian or Alaskan Native and non-Hispanic Black or African American populations. For a second year, COVID-19 was the third leading cause of death after heart disease and cancer.

**What are the implications for public health practice?**

Provisional death estimates provide an early signal about shifts in mortality trends. Provisional findings about increases in mortality for certain populations and for certain causes of death can guide public health policies and interventions.

## Footnotes

* NVSS provisional mortality data are available at https://wonder.cdc.gov. NVSS COVID-19 surveillance data are available at https://www.cdc.gov/nchs/nvss/deaths.htm.

† Based on death records received and processed as of April 12, 2022, for deaths occurring in the United States among U.S. residents. Data included in this analysis include >99% of deaths occurring in 2021.

§ The underlying cause of death is the disease or injury that initiated the train of morbid events leading directly to death.

¶ At the time of analysis, 0.2% of total NVSS deaths, and 0.2% of COVID-19 deaths, were among foreign residents and residents of U.S. territories.

** 45 C.F.R. part 46, 21 C.F.R. part 56; 42 U.S.C. §241(d); 5 U.S.C. §552a; 44 U.S.C. §3501 et seq.
